# Advanced Polymeric Scaffolds for Stem Cell Engineering and Regenerative Medicine

**DOI:** 10.3390/polym16182667

**Published:** 2024-09-22

**Authors:** João Carlos Silva, Frederico Castelo Ferreira

**Affiliations:** 1 Department of Bioengineering and iBB-Institute for Bioengineering and Biosciences, Instituto Superior Técnico, Universidade de Lisboa, Av. Rovisco Pais, 1049-001 Lisboa, Portugal; frederico.ferreira@tecnico.ulisboa.pt; 2 Associate Laboratory i4HB—Institute for Health and Bioeconomy, Instituto Superior Técnico, Universidade de Lisboa, Av. Rovisco Pais, 1049-001 Lisboa, Portugal

Polymeric scaffolds play a pivotal role in tissue engineering (TE) and regenerative medicine strategies, as they offer the possibility to closely mimic the architectural features of the native tissues’ extracellular matrix (ECM) and support cell performance both in vitro and in vivo, creating a favourable regenerative microenvironment [1]. Natural and synthetic polymers have been widely used in TE to fabricate scaffolds with defined structural, biological, mechanical and degradation properties to guide specific cellular behaviours (e.g., adhesion, migration, proliferation and differentiation) and meet the particular requirements for the regeneration of different target tissues [2,3]. Recent multidisciplinary developments in the design and engineering of new stimuli-responsive polymers and hybrid bioactive composites, as well as significant advances in scaffold biofabrication technologies (e.g., additive manufacturing—3D bioprinting and electrospinning), have increased the structural complexity and regenerative potential of polymer-based scaffolds [4,5]. This enhanced functionality achieved by state-of-the-art polymeric scaffolds strongly encourages their use in combination with stem cells towards the development of disruptive TE strategies and reliable in vitro disease models with high potential for clinical translation.

This Special Issue focuses on bringing together contributions covering the most recent and exciting approaches to the development of polymeric biomaterial scaffolds for regenerative medicine and disease modelling applications. This multidisciplinary publication comprises sixteen manuscripts, of which eleven are original research papers and five are review papers, from research groups all over the world. These contributions, which are briefly summarized below, cover a wide range of topics related to polymeric scaffolds, from novel bioinks for 3D bioprinting, engineered hydrogels for soft tissue repair, and drug delivery systems for anticancer therapies to the optimization of scaffolds’ structural and mechanical properties through the use of additives or following mathematical design and in silico modelling approaches.

Carvalho and colleagues [6] reviewed and discussed the potential of bone ECM non-collagenous proteins for bone and dental TE strategies. More specifically, the authors presented an overview of the different non-collagenous proteins found in bone ECM, highlighting their important role in regulating cellular processes such as adhesion, proliferation, osteogenic differentiation and angiogenic potential, all of which are crucial for successful bone TE strategies.

Hybrid composite scaffolds combining biological materials with synthetic polymers to achieve constructs with high bioactivity and appropriate mechanical properties hold great promise for regenerative medicine. Accordingly, Sawyer et al. [7] reviewed the use of a commercially available reinforced tissue matrix (RTM) device (OviTex), developed by embedding a polymer within a decellularized ovine-derived ECM, in several types of hernia repair treatments. The authors observed that the use of the RTM in hernia repair promoted wound healing, with no evidence of foreign body response, and provided structural tissue reinforcement, lowering the risk of hernia recurrence.

Electrically conductive polymeric scaffolds are attracting considerable interest for TE applications, particularly in strategies targeting the regeneration of electroactive tissues such as bone, skeletal muscle, neural, and cardiac tissue [4]. In this context, Bartoli et al. [8] presented an overview of the use of nanostructured carbon fillers (e.g., carbon nanotubes (CNTs) and graphene-related materials) to produce biopolymer composites with enhanced mechanical properties and electrical conductivity, particularly targeting TE applications. Accordingly, Sanjuan-Alberte and colleagues [9] developed conductive photocurable inks for two-photon polymerization (2PP) 3D printing by dispersing multi-walled CNTs (MWCNTs) within a gelatin methacrylate (GelMa) solution. The enhanced electrical properties of the formulated hydrogel inks were demonstrated through impedance spectroscopy and cyclic voltammetry. Moreover, the conductive material was shown to support the viability and growth of human induced pluripotent stem cell (iPSC)-derived cardiomyocytes. Finally, the authors were able to manufacture, for the first time, micron-sized conductive hydrogels via 2PP, highlighting the potential of their strategy for applications in TE and bioelectronics. Considering the current need to develop appropriate scaffolds and setups for the electrical stimulation of cells, Garrudo et al. [10] reviewed the existing systems based on poly(aniline):camphorsulfonic acid (PANI:CSA), one of the most well-studied electroconductive polymers, with a particular focus on applications for neural cell culture and differentiation.

The application of innovative biomaterials, namely electrospun nanofibers, nanoparticles (NPs), hydrogels and 3D printed scaffolds, in anticancer therapies and regenerative strategies after tumour resection is comprehensively discussed in a review paper by Zaszczyńska and colleagues [11]. Considering this, Ferrentino et al. [12] synthetized and characterized amphiphilic poly(ε-caprolactone)-poly(ethylene glycol) (PCL-PEG-PCL) triblock copolymers, which self-assembled in the form of core–shell micelles. The PCL-PEG-PCL core–shell NPs were characterized using dynamic light scattering and nuclear magnetic resonance, as well as in terms of their capacity for uptake by human colorectal carcinoma cells. Finally, the developed micelles were loaded with the hydrophobic molecule quercetin, and the anticancer properties of the quercetin-loaded NPs were demonstrated using a human colon cancer cell line (HCT 116 cells). In a different study addressing cancer, Banda Sánchez and colleagues [13] describe the selection and optimization of a bioink and bioprinting process for pancreatic tumour modelling. The ink, composed of plasma-loaded alginate/methylcellulose (Alg/MC), was characterized in terms of its viscoelastic behaviour, gelation kinetics and degree of recovery, showing suitability for 3D bioprinting. When combined with pancreatic tumour cells (PANC-1), the cell-laden bioink was able to generate 3D-bioprinted models featuring proper geometrical fidelity while also supporting the PANC-1 cells’ viability, typical phenotype and proliferation capacity.

The development of tissue-engineered vascular grafts (TEVGs) for the regeneration of small arteries remains an unmet clinical need. Nevertheless, recent approaches using polymeric scaffolds in vascular TE strategies have achieved highly promising results both in vivo and in vitro. Antonova et al. [14] developed a TEVG based on poly(3-hydroxybutyrate-co-3-hydroxyvalerate)/poly(ε-caprolactone) loaded with vascular endothelial growth factor (VEGF), basic fibroblast growth factor (bFGF) and stromal cell-derived factor 1α (SDF-1α) and surface-coated with heparin and illoprost (PHBV/PCL[VEGF-bFGF-SDF]^Hep/Ilo^). The developed TEVG was evaluated in a sheep carotid artery interposition model using biostable vascular prostheses of expanded poly(tetrafluoroethylene) as a control. The authors observed that the PHBV/PCL[VEGF-bFGF-SDF]^Hep/Ilo^ grafts showed a promising primary patency rate in comparison to the control, and the regenerated arteries presented high vascularization, full endothelialization and a multilayer hierarchical structure similar to that of the native blood vessels. Wang et al. [15] synthesized biodegradable and electrospinnable α-amino acid-substituted poly(organophosphazene) (PαAPz) polymers and subsequently fabricated and characterized PαAPz electrospun nanofibrous scaffolds for vascular TE applications. The authors were able to produce bead-free fibrous scaffolds, with average diameters of 100 to 300 nm, that supported the adhesion and spread of human smooth muscle cells (SMCs), bone marrow-derived mesenchymal stem/stromal cells (BM-MSCs) and iPSC-derived MSCs (iMSCs). Notably, in vitro studies evidenced that PαAPz nanofibers were able to promote the differentiation of iMSCs towards an SMC lineage, suggesting their potential for the fabrication of engineered functional vascular tissues.

Collagen membranes are the most widely used biomaterial for guided bone regeneration procedures in dentistry and periodontology. However, their fast degradation kinetics limits the success of these therapeutic approaches. To understand whether the collagen’s origin might have an important effect on the biodegradation process, Vallecillo-Rivas et al. [16] studied the degradation patterns of five commercially available collagen membranes (Biocollagen, Heart, Evolution X-fine, CopiOs and Parasorb Resodont) of different origins in three different solutions (phosphate-buffered saline (PBS), bacterial collagenase from *Clostridium histolyticum* and porcine trypsin). Overall, the Evolution X-fine collagen membrane derived from porcine pericardium was the least affected when exposed to the three different solutions, while Biocollagen and Parasorb Resodont (both of equine origin) presented the highest degradation rates. Moreover, the bacterial collagenase solution was the most aggressive medium, with four out of the five membranes (with the exception of Evolution X-fine) being totally degraded after 7 days of exposure.

Focusing on the search for an appropriate biomaterial for the repair of dura mater (DM) defects, Lipovka and colleagues [17] compared the mechanical properties of bacterial nanocellulose (BNC) impregnated with chitosan nanoparticles (Novochizol^TM^) and the antibiotic vancomycin with those of native BNC and with those of cadaveric DM (preserved in formalin) and human native DM. The authors reported that the BNC + Novochizol^TM^ + vancomycin achieved strength properties similar to those of DM (cadaveric and fresh). Moreover, the strength features were significantly improved by the addition of Novochizol^TM^ to the BNC and could be modulated by varying the polymer thickness.

The management and functional regeneration of articular cartilage and osteochondral (OC) defects are still among the most challenging clinical issues in orthopaedics. Biodegradable and non-biodegradable polymeric scaffolds have been widely used in cartilage regeneration and replacement strategies, respectively. Considering a cartilage replacement approach, Duque-Ossa et al. [18] developed a solid/liquid triborheological system to recreate the conditions in human synovial joints. The system was used to evaluate the potential of polyvinyl alcohol (PVA) hydrogels for articular cartilage replacement under physiological conditions and using hyaluronic acid (HA) as a lubricant. The pressures applied during the tests simulated the impact experienced daily in these joints. The PVA hydrogel characterization results showed an equilibrium water content of around 83%, which is very similar to that of native articular cartilage (80%), and a compression Young’s modulus of 2.26 MPa. Triborheological tests confirmed a coefficient of friction in the range of 0.1–0.4, which is compatible to that of natural cartilage (0.1) and supports the hypothesis that PVA hydrogels lubricated with HA might represent a promising mechanical alternative for future tissue replacement strategies. In another study focused on cartilage repair, Fu and colleagues [19] optimized the formulations of injectable hydrogels based on tyramine (TA)-functionalized HA and dextran (Dex) towards improved chondrocyte growth and matrix synthesis. Based on the high biocompatibility, improved mechanical properties, and enhanced glycosaminoglycan and collagen production, the authors observed that the 5% *w*/*v* hybrid hydrogels produced with a 25%/75% HA/Dex ratio were the most promising constructs for cartilage TE. Another study performed by Cordeiro et al. [20] combined PCL with cellulose from different sources (commercially available: microcrystalline and methylcellulose, and cellulose obtained from agro-industrial residues) to fabricate scaffolds for cartilage TE using Fused Deposition Modelling (FDM). The produced composite scaffolds were characterized in comparison to pure PCL in terms of their morphology, chemical and mechanical properties, enzymatic degradation profile and cytocompatibility. Overall, the results suggest that cellulose (particularly methylcellulose) can be incorporated into PCL scaffolds to produce scaffolds with compressive properties closer to those of native cartilage and enhance the proliferation of human dental pulp stem/stromal cells. In a different study focused on OC repair, Marcelino et al. [21] presented a novel method for designing and manufacturing scaffolds with a mathematically defined curvature (based on the geometry of a sphere) able to mimic the native OC tissue shape. The printability of the scaffolds using poly lactic acid (PLA) and FDM equipment was evaluated, and a limit sphere radius was defined to ensure high printing fidelity without defects, as confirmed via scanning electron microscopy and micro-computed tomography. The mechanical behaviour of the produced curved scaffolds was evaluated experimentally under compressive tests and by means of finite element modelling, which allowed for the identification of the scaffold regions subjected to higher loads. Finally, the authors highlighted the potential of combining numerical modelling with experimental approaches towards the development of improved biomimetic scaffolds for OC TE strategies.

In conclusion, this Special Issue provides the readers of *Polymers* with an outstanding and multidisciplinary array of exciting recent research and literature reviews on the development of advanced polymer scaffolds for regenerative medicine applications, highlighting their potential for the development of more affordable and sustainable personalized therapies.
